# Knowledge, Attitude, and Practice Regarding Periodontal and Dental Diseases During Pregnancy Among Obstetricians and Dentists in King Saud University Medical City

**DOI:** 10.7759/cureus.47098

**Published:** 2023-10-16

**Authors:** Hani AlHalal, Rawan M Albayyat, Nawaf K Alfhaed, Omar Fatani, Bader Fatani

**Affiliations:** 1 Obstetrics and Gynecology, King Saud University, College of Medicine, King Saud University Medical City, Riyadh, SAU; 2 Medicine, King Saud University, College of Medicine, Riyadh, SAU; 3 Dentistry, College of Dentistry, King Saud University, Riyadh, SAU; 4 Obstetrics and Gynecology, King Saud University Medical City, Riyadh, SAU

**Keywords:** prenatal, oral health, periodontal disease, pregnancy, dental disease

## Abstract

Background: Providing effective dental treatment during pregnancy is related to controlling oral diseases and helps maintain a healthy oral cavity. One of the possible treatment options for endodontic disease is to treat the odontogenic infection, maintain a healthy oral environment, and minimize the expected complications that can occur later in pregnancy or during the postpartum period. Sufficient awareness among obstetricians and dentists is essential to delivering appropriate preventive and curative care to pregnant patients.

Objective: The aim of this cross-sectional study is to assess knowledge, attitudes, and behaviors regarding oral health care and providing dental treatment among King Saud University Medical City obstetricians and dentists during pregnancy and the association of dental and periodontal disease with adverse pregnancy outcomes.

Methods: This study was conducted in Saudi Arabia, Riyadh, from December 2022 to June 2023 (six months). The data were collected from 381 participants in Riyadh, Saudi Arabia, from January 2023 to June 2023. The study targeted male and female general dentists and obstetricians living in Riyadh, Saudi Arabia, and excluded those who are not dentists and obstetricians or not living in Riyadh, Saudi Arabia.

Result: Out of a total of 381 completed responses, 281 (73.8%) of the participants were dentists, while 100 (26.2%) were obstetricians. It was observed that the majority of the participants agreed that oral health is a part of prenatal care, while the minority did not agree (0.3%). Of the 281 (73.8%) respondents, most of them reported periodontal disease can cause preterm birth, low birth weight, and gestational diabetes (73.2%), while only 0.5% answered no. Two hundred and seventy-eight of the participants answered that it is essential to consult with an obstetrician before treating pregnant patients.

Conclusion: The overall knowledge of dentists and obstetricians was adequate regarding dental disease during pregnancy. However, more education should be assessed and continuously improved regarding the treatment options, as well as future recommendations regarding the medications used in dental clinics and awareness programs promoting dental health care in pregnant patients.

## Introduction

Pregnancy involves multiple hormonal, physical, immunologic, nutritional, and behavioral changes that greatly affect almost all organ systems, including the oral cavity [1,2]. Gingivitis, periodontal infection, and changes in both the hard and soft tissues are the main oral disorders connected to pregnancy [3]. In some cases, morning sickness, vomiting, and hyperemesis gravidarum can cause enamel erosion that results in loss, particularly on the vestibular surface [4]. Fluctuations in diet and frequency of eating, combined with hormonal changes, can raise the risk of tooth decay [5,6]. The most prevalent oral conditions seen in pregnant patients are, by far, gingivitis and periodontitis [2]. Approximately 60% to 75% of pregnant women develop pregnancy gingivitis [4]. The rising levels of both progesterone and estrogen throughout pregnancy have been discovered to influence the development of periodontal disease and wound healing [7]. Both of these hormones cause a reduction in immunological response and an increase in gingival vascularization [8]. Additionally, research shows that certain bacteria grow during pregnancy, such as Prevotella intermedia and Prevotella melaninogenica [9,10]. These germs make the gingival inflammation worse and make the gums more prone to bleeding [9,10]. As a result, even with very modest plaque levels, pregnant patients experience severe gingival irritation [2]. Effective endodontic treatment during pregnancy contributes to the prevention of oral illnesses and promotes oral health [11]. Controlling the progression of odontogenic infection, maintaining a healthy oral environment, and minimizing the expected complications that may arise later in pregnancy or during the postpartum period are some of the available treatments for endodontic disease [12]. To provide adequate preventative and therapeutic care to pregnant patients and obstetricians, dentists must acknowledge and understand this condition to a sufficient degree. Additionally, pregnancy is not a reason to postpone endodontic treatment, but its management requires certain considerations, such as adjusting the timing and dosing of treatments and other interventions that need to be provided [13,14]. An accumulation of evidence suggests a link between periodontal disease, also known as inflammatory gum disease, and adverse pregnancy outcomes such as preterm birth, low birth weight, and preeclampsia [15]. A significant portion of the dentistry community thinks that painkiller use, amalgam repair, and periodontal surgery can be hazardous to pregnant women [4]. Given the safety of endodontic therapy during pregnancy, general dentists are frequently hesitant to provide endodontic treatment to pregnant patients [16]. Studies carried out in Saudi Arabia [17] and Nigeria [18] show that the knowledge delivered among dental practitioners is not sufficient to treat pregnant or nursing women. In a Swiss study assessing the knowledge, attitudes, and practices (KAP) of dentists in the Dominican Republic regarding the prescription of antibiotics to pregnant or nursing women, they came to the conclusion that most dentists get their information from their undergraduate education or specialized websites rather than from the scientific literature [19]. An Indian study assessing knowledge, attitude, and practice behaviors among obstetricians and dental practitioners revealed that dental treatment during the pregnancy phase shows some alteration from scientific literature recommendations [20]. There is a severe lack of systematic data to assess obstetricians' and dentists' knowledge, attitudes, and practices regarding maternal oral health care during pregnancy and its impact on the course of pregnancies. To the best of our knowledge, no study has been published in Saudi Arabia that evaluated the depth of information held by healthcare professionals regarding the maintenance of dental health throughout pregnancy and the relationship between oral health and pregnancy outcomes. The aim of this cross-sectional study is to assess the knowledge, attitudes, and practices of obstetricians and dentists in Saudi Arabia with reference to dental health treatment during pregnancy.

## Materials and methods

This study was conducted in Saudi Arabia, Riyadh, from December 2022 to June 2023 (six months) and was an observational, quantitative, cross-sectional study that focused on obstetricians and dentists at King Saud University Medical City, Riyadh, KSA. The study included obstetricians and dentists of both genders at King Saud University Medical City and excluded obstetricians and dentists from non-King Saud University Medical City. The sample size of 100 of each specialty (OBGYN and dentistry) was determined using the equation n=Z2pq/d2, where n is the desired sample size, Z is the standard normal deviation set at 1.96 for a 95% confidence interval, p is the proportion, q is 1-p, and d is the precision of ±5%. With a non-response rate of 20%, the total number of participants required was 120. The study examined sociodemographic characteristics like age, gender, years in practice, place of practice, and specialty. It employed self-administered closed-ended questionnaires to assess participants' knowledge of gingival/periodontal infection during pregnancy [2,5,11,16,21], as well as their source of information [5,11,16,20], preferences for dental treatment [11,16,19,21,22], procedures, and positioning [2,5,11,16]. A structured interview was used as a study tool based on previous studies in Saudi Arabia and elsewhere. The questionnaire consisted of two sections: socio-demographic data and questions related to participants' subjects of interest. The collected data were analyzed using SPSS 29.0 version statistical software. Descriptive statistics were done using bivariate analysis with statistical tests such as chi-square, t-tests, and correlation analysis. While frequencies and percentages were used when variables were categorical, statistical significance was indicated by a p-value of <0.05, and a 95% confidence interval was used to report precision. The study has been approved by the institutional research board (IRB) at the College of Medicine, King Saud University, Riyadh, Saudi Arabia, ethical approval number (E-22-7396). Consent was obtained in English or Arabic from each participant. Participants were informed of their right to discontinue at any point. All the data collected were kept confidential.

## Results

A total of 381 responses were completed; most of the participants were dentists, 281 (73.8%), and 100 (26.2%) were obstetricians. When considering the years in practice, approximately half of them were between one and ten years old, and about one-half of the participants were between 31 and 40 years old (47.2%). Of these, 210 (55.1%) were males, and 171 (44.9%) were females (Table 1).

**Table 1:** Bio-demographic data of the participants.

Bio-demographic	Number	Percent
Age (in years)		
≤30	130	34.1
31–40	180	47.2
41–50	52	13.6
≥50	19	5.0
Gender		
Male	210	55.1
Female	171	44.9
Specialty		
Dentist	281	73.8
Obstetrician	100	26.2
Years in practice		
Less than 1 year	3	0.8
1–10	180	47.2
11–20	148	38.8
21–30	47	12.3
31–40	3	0.8

Table 2 presents the knowledge, attitude, and behavior levels regarding oral health care and providing dental treatment during pregnancy among KSUMC dentists during pregnancy. The participating dentists were 281 (73.8%).

**Table 2:** Knowledge, attitude, and behaviors levels regarding oral health care and providing dental treatment during pregnancy among KSUMCs dentists during pregnancy. CBCT: cone beam computed tomography, IOPA: intraoral periapical, OPG: orthopantomogram, RVG: radiovisiography.

Question	Frequency	Percent
Can periodontal disease cause preterm birth, low birth weight, and gestational diabetes?		
Yes	279	73.2
No	2	0.5
Do hormonal changes hinder the body's ability to repair soft tissue?		
Yes	263	69
No	18	4.7
Have you ever performed dentistry treatments for pregnant patients?		
Yes	234	61.4
No	47	12.3
Do you think that providing pregnant women with health instructions is essential prior to any treatments?		
Yes	275	72.2
No	6	1.6
Do you think that it is essential to consult with an obstetrician before treating pregnant patients?		
Yes	278	73
No	3	0.8
Do you think that you have enough required knowledge about dental considerations during pregnancy?		
Yes	187	49.1
No	94	24.7
What knowledge do you rely on for prescribing antibiotics to pregnant women?		
Knowledge acquired during undergraduate studies	65	17.1
Knowledge of scientific literature	211	55.4
Specialized websites	5	1.3
Safest trimester of pregnancy for provision of dental treatment		
First trimester	11	2.9
Second trimester	268	70.3
Third trimester	2	0.5
Preference and use of local anesthesia		
Lidocaine hydrochloride with adrenaline of 1:100,000	244	64
Lidocaine hydrochloride with adrenaline of 1:100,000, prilocaine	18	4.7
Mepivacaine	15	3.9
Prilocaine	4	1
Preference and use of dental X-ray		
CBCT	1	0.3
Conventional IOPA	92	24.1
OPG, RVG	21	5.5
OPG	9	2.4
RVG	158	41.5
What is the right position in the dental unit in the last trimester of pregnancy?		
Lying to the left supine	176	46.2
Lying to the right	105	27.6
Which analgesic is safe during pregnancy?		
Acetaminophen	258	67.7
Acetaminophen codeine	20	5.2
Indomethacin	3	0.8
What is the reason for not prescribing tetracycline during pregnancy?		
Delayed delivery	61	16
Fetal bradycardia	111	29.1
Tooth discoloration	109	28.6
What are the side effects of using narcotics during pregnancy?		
Cleft of lip and palate	16	4.2
Delayed delivery	22	5.8
Preterm delivery	130	34.1
Respiratory depression	113	29.7
Obstetricians should refer the pregnant patient to the dentist to observe any oral clinical findings		
Yes	279	73.2
No	1	0.3
Do pregnant women need for the CDE program?		
Yes	274	71.9
No	7	1.8

Of the 281 (73.8%) respondents, most of them reported periodontal disease can cause preterm birth, low birth weight, and gestational diabetes (73.2%). The majority of the dentists (69%) answered that hormonal changes hinder the body's ability to repair soft tissue, while the minority (4.7%) answered that hormonal changes do not hinder the body's ability to repair soft tissue. When the dentists were asked if they had ever performed dentistry treatments for pregnant patients, most of them answered yes (61.4%). Regarding whether providing pregnant women with health instructions is essential prior to any treatments, 275 (72.2%) answered yes. Two hundred and seventy-eight of the participants answered that it is essential to consult with an obstetrician before treating pregnant patients. Half of the participating dentists thought that they had enough knowledge about dental considerations during pregnancy (49.1%), while 24.7% thought that they did not have enough knowledge. More than half of the participants (55.4%) relied on knowledge of scientific literature, 17.1% relied on knowledge acquired during undergraduate studies, and 1.3% relied on specialized websites. The vast majority (70.3%) answered that the second trimester is the safest trimester of pregnancy for the provision of dental treatment, and only 0.5% answered that the safest trimester is the third trimester. More than half of the participants (64%) chose lidocaine hydrochloride with adrenaline at 1:100,000, while only 1% chose prilocaine. Radiovisiography (RVG) is the most common dental X-ray chosen (41.5%). On the other hand, cone beam computed tomography (CBCT) has been chosen by only one participant. More than half of the participants (46.2%) reported that the right position is lying to the left. Lying to the right, 27.6% of the participants (67.7%) have chosen acetaminophen as the safe analgesic during pregnancy, while only 0.8% reported that indomethacin is the safe analgesic during pregnancy. Fetal bradycardia (29.1%), tooth discoloration (28.6%), and delayed delivery (16%) are the top three reasons for not prescribing tetracycline during pregnancy. Most of the dentists (34.1%) have chosen preterm delivery as the side effect of using narcotics during pregnancy, while 4.2% have chosen clefts of the lip and palate as the side effect.

Regarding the fact that the obstetrician should refer the pregnant patient to the dentist on observing any oral clinical findings, 73.2% of the participants, which is the vast majority, have agreed, while only 0.3% have not. Regarding whether pregnant women need the CDE program, 71.9% answered yes, they need the CDE program; only 1.8% answered no, they do not need the CDE program.

There was a statistically significant difference (p-value = 0.00) observed for a knowledge-based question that assessed if hormonal changes hinder the body's ability to repair soft tissue. This sentence was agreed with by 80% of the younger 30-year-old age group, 97.4% of the 31-40 age group, 100% of the 41-50 age group, and 100% of the over-50 age group. Also, statistical differences were observed (p-value = 0.00) when the participants were asked if they had ever performed dentistry treatments for pregnant patients. This statement was agreed with by 58.6% of the younger 30-year-old age group, 89.7% of the 31-40 age group, 94.7% of the 41-50 age group, and 100% of the over-50 age group. In addition, there is a statistical difference (p-value = 0.00) in the responses when the participants have been asked if they have performed dentistry treatments for pregnant patients. This statement was agreed with by 51.4% of the younger 30-year-old age group, 64.7% of the 31-40 age group, 86.8% of the 41-50 age group, and 100% of the over-50 age group. Whereas there were no statistically significant differences observed among the different age groups and their responses to the attitude and practice of the other questions (Table 3).

**Table 3:** Chi-squared test for associations among dentists different age groups of the and knowledge, attitude, and practice questions.

		Age				P-value
		≥30	31–40	41–50	≤50	
Hormonal changes hinders the body's ability for repair of soft tissue?	Yes	56	152	38	17	0.000
		80.0%	97.4%	100.0%	100.0%	
	No	14	4	0	0	
		20.0%	2.6%	0.0%	0.0%	
Have you ever performed dentistry treatments for pregnant patients?	Yes	41	140	36	17	0.000
		58.6%	89.7%	94.7%	100.0%	
	No	29	16	2	0	
		41.4%	10.3%	5.3%	0.0%	
Do you think that providing pregnant women with health instructions are essential prior to any treatments?	Yes	68	153	38	16	0.536
		97.1%	98.1%	100.0%	94.1%	
	No	2	3	0	1	
		2.9%	1.9%	0.0%	5.9%	
Do you think that it is essential to consult with a obstetrician before treating pregnant patients?	Yes	70	153	38	17	0.488
		100.0%	98.1%	100.0%	100.0%	
	No	0	3	0	0	
		0.0%	1.9%	0.0%	0.0%	
Do you think that you have enough required knowledge about dental considerations during pregnancy?	Yes	36	101	33	17	0.000
		51.4%	64.7%	86.8%	100.0%	
	No	34	55	5	0	
		48.6%	35.3%	13.2%	0.0%	
Which analgesic is safe during pregnancy?	Acetaminophen	62	142	37	17	0.642
		88.6%	91.0%	97.4%	100.0%	
	Acetaminophen codeine	7	12	1	0	
		10.0%	7.7%	2.6%	0.0%	
	Indomethacin	1	2	0	0	
		1.4%	1.3%	0.0%	0.0%	
Obstetrician should refer the pregnant patient to the dentist on observing any oral clinical findings?	Yes	68	156	38	17	0.381
		98.6%	100.0%	100.0%	100.0%	
	No	1	0	0	0	
		1.4%	0.0%	0.0%	0.0%	

There were no statistically significant differences observed among the different genders and their responses to the attitude and practice of the other questions (Table 4).

**Table 4:** Chi-squared test for associations among dentists different gender groups of the and knowledge, attitude, and practice questions.

		Gender		P-Value
		Male	Female	
Hormonal changes hinders the body's ability for repair of soft tissue?	Yes	141	122	0.125
		91.6%	96.1%	
	No	13	5	
		8.4%	3.9%	
Have you ever performed dentistry treatments for pregnant patients?	Yes	126	108	0.471
		81.8%	85.0%	
	No	28	19	
		18.2%	15.0%	
Do you think that providing pregnant women with health instructions are essential prior to any treatments?	Yes	151	124	0.811
		98.1%	97.6%	
	No	3	3	
		1.9%	2.4%	
Do you think that it is essential to consult with a obstetrician before treating pregnant patients?	Yes	152	126	0.678
		98.7%	99.2%	
	No	2	1	
		1.3%	0.8%	
Do you think that you have enough required knowledge about dental considerations during pregnancy?	Yes	106	81	0.372
		68.8%	63.8%	
	No	48	46	
		31.2%	36.2%	
Which analgesic is safe during pregnancy?	Acetaminophen	140	118	0.811
		90.9%	92.9%	
	Acetaminophen Codeine	12	8	
		7.8%	6.3%	
	Indomethacin	2	1	
		1.3%	0.8%	
Obstetrician should refer the pregnant patient to the dentist on observing any oral clinical findings?	Yes	153	126	0.272
		100.0%	99.2%	
	No	0	1	
		0.0%	0.8%	

Table 5 represents the opinions of dentists regarding antibiotic treatment during pregnancy. Participants were allowed to choose one option. Most of the participants (87%) chose endodontics in teeth with symptomatic irreversible pulpitis and with or without symptomatic apical periodontitis, which is the case that needs antibiotics, followed by endodontics in teeth with pulp necrosis and with or without symptomatic apical periodontitis, which represents 41%.

**Table 5:** Opinions of dentists regarding antibiotics treatment during pregnancy.

For which treatments do you prescribe antibiotics to pregnant patients?	Frequency	Percent
Endodontics in teeth with symptomatic irreversible pulpitis and with/without symptomatic apical periodontitis	87	30.96%
Endodontics in teeth with pulp necrosis and with/without symptomatic apical periodontitis	41	14.59%
Endodontics in teeth with necrosis and periapical granuloma	37	13.16%
Endodontics in teeth with pulp necrosis and acute apical abscess with/without systemic involvement	35	12.45%
Simple exodontia	4	1.42%
Pericoronitis	28	9.96%
Cellulitis	19	6.76%
Scaling and root planning	9	3.20%
Periodontal abscess	21	7.47%

Table 6 lists the various dental procedures that dentists advised participants to get while they were pregnant. Participating dentists were allowed to choose several options rather than only one, which resulted in the main type of treatment they thought their pregnant patients could receive being an examination.

**Table 6:** Types of dental treatment participants advised by dentists to be received during pregnancy.

Types of dental treatment participants advised to be received during pregnancy?	Frequency	Percent
Routine cleaning, fillings/crowns	1	0.3
Examination	98	25.7
Examination, fillings/crowns	55	14.4
Examination, periodontal (gum) treatment	15	3.9
Examination, outline cleaning	96	25.2
Fillings/crowns, others	1	0.3
Other	15	3.9

Table 7 displays the responses of the gynecologists to the questions aimed at evaluating their knowledge, attitude, and behavior levels regarding the association between oral health and pregnancy. The participating obstetricians are 100 (26.2%) out of 381 participants. In this section, we will discuss the answers of obstetricians to questions related to them. It was observed that the majority of the participants agreed that oral health is a part of prenatal care, while the minority did not agree (0.3%). Regarding periodic oral examinations on a regular basis, the majority of the gynecologists (23.1%) agreed on this information during the pregnancy. The greatest proportion (24.1%) of the respondents agreed on the importance of oral health and the referral of pregnant women to dental resources. Almost more than three-quarters (24.1%) of the participating gynecologists have agreed that eating habits increase the risk of dental caries and periodontal diseases. Regarding whether pregnancy increases the likelihood of gingival inflammation, the majority of the gynecologists (25.5%) stated yes, it increases the likelihood of gingival inflammation. Regarding whether there is a possible connection between the health of the teeth and gums and pregnancy, almost all the gynecologists stated yes, there is a connection. 24.1% of gynecologists believe that gingival or periodontal inflammation can affect the outcome of pregnancy. 24.1% of the gynecologists thought periodontal disease could lead to preterm labor and/or low birth weight, while a small percentage stated no. 25.2% reported advising their pregnant patient to visit the dentist during their pregnancy. However, most of the gynecologists (23.1%) were not advising patients to delay treatment until after pregnancy. Regarding whether their patients reported bleeding gums or tooth mobility during pregnancy, yes was the most common answer to this question, and 23.6% of the participants agreed on this situation. 24.4% of them believe it is safe to use regular local anesthetic solutions containing vasoconstrictors for pregnant patients. 25.2% chose to advise patients to include periodontal evaluation as part of their prenatal care. The vast majority (25.7%) of the participating gynecologists thought pregnancy increased the tendency for the gums to bleed, swell, or be red. 24.4% of them thought tooth and gum problems could affect the outcome of pregnancy. More than half of the participants, or 24.9%, think that the developing baby will draw calcium from your teeth. Almost all of the participating obstetricians think that there is a possible connection between the health of the teeth and gums and pregnancy, except for one obstetrician. 25.5% of the participating obstetricians believe that periodontal diseases can be treated safely during pregnancy with a procedure called scaling and root planning, except for three obstetricians.

**Table 7:** Knowledge, attitude, and behaviours levels regarding oral health care and providing dental treatment during pregnancy among KSUMCs obstetricians during pregnancy.

Question	Frequency	Percent
Oral health as a part of prenatal care		
Agree	86	22.6
Disagree	1	0.3
Not sure	13	3.4
Periodic oral examination on a regular basis		
Agree	88	23.1
Disagree	1	0.3
Not sure	11	2.9
Importance of oral health and referral of pregnant women to dental resources		
Agree	92	24.1
Disagree	0	0.0
Not sure	8	2.1
Newfound eating habits increase the risk of dental caries and periodontal diseases		
Agree	92	24.1
Disagree	1	0.3
Not sure	7	1.8
Do you agree that pregnancy increases the likelihood of gingival inflammation?		
Yes	97	25.5
No	3	0.8
Do you think that there is a possible connection between the health of the teeth and gum and pregnancy?		
Yes	99	26.0
No	1	0.3
Do you believe that gingival/periodontal inflammation can affect the outcome of pregnancy?		
Yes	92	24.1
No	8	2.1
Do you think periodontal disease can lead to preterm labour and/or low birth weight?		
Yes	92	24.1
No	8	2.1
Advising patients to visit the dentist during pregnancy		
Yes	98	25.7
No	2	0.5
Advising patient to delay visit to the dentist until after pregnancy		
Yes	12	3.1
No	88	23.1
Have your patients reported bleeding gums or tooth mobility during pregnancy?		
Yes	90	23.6
No	10	2.6
Do you believe it is safe to use regular local anesthetic solutions containing vasoconstrictors for pregnant patients?		
Yes	93	24.4
No	7	1.8
Advising patients to include periodontal evaluation as part of their prenatal care		
Yes	96	25.2
No	4	1.0
Do you think pregnancy increases the tendency for the gums to bleed, swell, or be red?		
Yes	98	25.7
No	2	0.5
Do you think tooth and gum problems could affect the outcomes of pregnancy?		
Yes	93	24.4
No	7	1.8
Do you believe in the statement ‘a tooth for a baby?		
Yes	85	22.3
No	15	3.9
Do you believe calcium will be drawn out of your teeth by the developing baby?		
Yes	95	24.9
No	5	1.3
Do you think that there is a possible connection between the health of the teeth and gums and pregnancy?		
Yes	99	26.0
No	1	0.3
Do you believe that periodontal diseases can be treated safely during pregnancy with a procedure called scaling and root planning?		
Yes	97	25.5
No	3	0.8

Participating obstetricians were allowed to choose several options rather than only one, and the main types of treatment they thought their pregnant patients could receive were examinations (Table 8).

**Table 8:** Types of dental treatment participants advised by obstetricians to be received during pregnancy.

Types of dental treatment participants advised to be received during pregnancy	Frequency	Percent
Examination	4	1.0
Examination, filling and crown	3	0.8
Examination, periodontal (gum) treatment	8	2.1
Examination, routine cleaning	78	20.5
Routine cleaning, periodontal (gum) treatment, filling and crown	7	1.8

There was a statistically significant difference (p-value = 0.032) observed for a knowledge-based question that assessed whether the participants believed in the statement ‘a tooth for a baby'. 91.7% of the younger 30-year-old age group, 66.7% of the 31-40 age group, 85.7% of the 41-50 age group, and 100% of the over-50 age group agreed with this sentence. Also, statistical differences (p-value = 0.002) were observed when the participants were asked if they believed calcium would be drawn out of their teeth by the developing baby. 100% of the younger 30-year-old age group, 91.7% of the 31-40 age group, and 85.7% of the 41-50 age group agreed. While 50% agreed and 50% disagreed with this sentence for the over-50 age group, there were no statistically significant differences observed among the different age groups and their responses to the attitude and practice of the other questions (Table 9).

**Table 9:** Chi-squared test for associations among obstetricians different age groups of the and knowledge, attitude, and practice questions.

Question		Age				P-value
		≥30	31-40	41-50	≤50	
Oral health as a part of prenatal care	Agree	56	17	11	2	0.101
		93.3%	70.8%	78.6%	100.0%	
	Disagree	1	0	0	0	
		1.7%	0.0%	0.0%	0.0%	
	Not sure	3	7	3	0	
		5.0%	29.2%	21.4%	0.0%	
New found eating habits increase risk to dental caries and periodontal diseases	Agree	59	19	12	2	0.096
		98.3%	79.2%	85.7%	100.0%	
	Disagree	0	1	0	0	
		0.0%	4.2%	0.0%	0.0%	
	Not Sure	1	4	2	0	
		1.7%	16.7%	14.3%	0.0%	
Advising patients to visit dentist during pregnancy	Yes	59	26	11	2	0.728
		96.7%	100.0%	100.0%	100.0%	
	No	2	0	0	0	
		3.3%	0.0%	0.0%	0.0%	
Do you think tooth and gum problems could affect outcomes of pregnancy?	Yes	57	20	14	2	0.170
		95.0%	83.3%	100.0%	100.0%	
	No	3	4	0	0	
		5.0%	16.7%	0.0%	0.0%	
Do you believe in the statement ‘a tooth for a baby?	Yes	55	16	12	2	0.032
		91.7%	66.7%	85.7%	100.0%	
	No	5	8	2	0	
		8.3%	33.3%	14.3%	0.0%	
Do you believe calcium will be drawn out of your teeth by the developing baby?	Yes	60	22	12	1	0.002
		100.0%	91.7%	85.7%	50.0%	
	No	0	2	2	1	
		0.0%	8.3%	14.3%	50.0%	
Do you think that there is a possible connection between the health of the teeth and gums and pregnancy?	Yes	59	24	14	2	0.879
		98.3%	100.0%	100.0%	100.0%	
	No	1	0	0	0	
		1.7%	0.0%	0.0%	0.0%	

There were no statistically significant differences observed among the different genders and their responses to the attitude and practice of the other questions (Table 10).

**Table 10:** Chi-squared test for associations among obstetricians' different gender groups of the knowledge, attitude, and practice questions.

Question		Gender		P-value
		Male	Female	
Oral health as a part of prenatal care	Agree	48	38	0.488
		85.7%	86.4%	
	Disagree	0	1	
		0.0%	2.3%	
	Not sure	8	5	
		14.3%	11.4%	
Newfound eating habits increase the risk of dental caries and periodontal diseases	Agree	50	42	0.458
		89.3%	95.5%	
	Disagree	1	0	
		1.8%	0.0%	
	Not sure	5	2	
		8.9%	4.5%	
Advising patients to visit the dentist during pregnancy	Yes	59	39	0.087
		100.0%	95.1%	
	No	0	2	
		0.0%	4.9%	
Do you think tooth and gum problems could affect the outcomes of pregnancy?	Yes	50	43	0.101
		89.3%	97.7%	
	No	6	1	
		10.7%	2.3%	
Do you believe in the statement 'a tooth for a baby'?	Yes	48	37	0.821
		85.7%	84.1%	
	No	8	7	
		14.3%	15.9%	
Do you believe calcium will be drawn out of your teeth by the developing baby?	Yes	54	41	0.460
		96.4%	93.2%	
	No	2	3	
		3.6%	6.8%	
Do you think that there is a possible connection between the health of the teeth and gums and pregnancy?	Yes	55	44	0.373
		98.2%	100.0%	
	No	1	0	
		1.8%	0.0%	

## Discussion

A previous study by Hashim et al. aimed to investigate the knowledge of gynecologists regarding oral health care during pregnancy and the role of periodontal disease in these cases. The study demonstrated that the majority of participants (over 90%) recognized that pregnancy can increase the chances of experiencing gingival inflammation. Likewise, a significant percentage (95.4%) of gynecologists acknowledged the positive correlation between oral health and pregnancy. Furthermore, 75.9% believed that inflammation of the gums and periodontal tissues can impact the outcome of pregnancy, while 67.6% agreed that periodontal disease could potentially result in preterm labor or low birth weight [2]. In correlation, our study showed that the majority of the gynecologists (25.5%) stated that pregnancy increases the likelihood of gingival inflammation. Almost all gynecologists state that there is a connection between the health of the gums and teeth and pregnancy. Moreover, 24.1% of gynecologists believe that gingival or periodontal inflammation can affect the outcome of pregnancy. The same participants also thought that it could lead to preterm labor and/or low birth weight. Most of the participants in our study stated that it is essential to consult with an obstetrician before treating pregnant patients. However, according to the Zanata et al. study, 27.8% of obstetricians responded that there was no need for previous consultation before any dental procedure [5]. Moreover, according to Zanata et al. [5], most of the dentists (41%) recommended the use of lidocaine without epinephrine (plain). This shows a difference in comparison to our study, in which more than half of the participants (64%) chose lidocaine hydrochloride with adrenaline at 1:100,000. According to Aragoneses et al. [19], knowledge acquired during undergraduate studies was the most chosen answer in terms of prescribing antibiotics to pregnant women. However, in our study, knowledge of scientific literature was the most chosen answer. According to our study, 25.2% reported advising their pregnant patients to visit the dentist during their pregnancy. However, most of the gynecologists (23.1%) were not advising patients to delay treatment until after pregnancy. On the other hand, the study by Al-Habashneh et al. [21] showed that 49.7% advised visiting a dentist during pregnancy. However, the majority advised delaying dental visits after pregnancy. Nepal study findings reveal that general dental practitioners realize the safety of endodontic treatment for pregnant women. However, they have inadequate knowledge regarding the safety of irrigants and radiographic exposure for pregnant women [11]. A Brazilian study evaluating the knowledge of obstetricians and dentists showed that dental treatment during the pregnancy phase still shows deviations from the scientific literature [5]. Another UAE study demonstrated that obstetricians have a relatively high level of knowledge. However, there are some misunderstandings regarding the provision of dental treatment during pregnancy [2]. The study by Al-HabashnehJordan et al. shows that the knowledge and awareness of physicians about gingival conditions and preterm birth are generally poor. Results from this survey showed inadequate awareness of the significance of oral health in maternal care and its connection to the infant’s overall health [21].

Limitations

The current limitation of the study was that the sample of pregnant women was not asked if they had an existing medical condition or if they just had a poor oral hygiene condition.

## Conclusions

Overall knowledge of dentists and obstetricians was adequate regarding dental disease during pregnancy. The vast majority of obstetricians agreed on the relationship between periodontal disease and pregnancy outcomes. On the contrary, the majority of dentists acknowledge that the body's ability to repair soft tissue, particularly that in the oral cavity, is impaired by hormonal fluctuations. However, more education should be assessed and continuously improved regarding the treatment options as well as future recommendations regarding the medications used for pregnant patients in dental clinics. For the sake of the mother's and the newborn's health, medical providers should follow a set of guidelines regarding prenatal and perinatal dental care. Moreover, this study demonstrates that dentists and obstetricians have poor communication and referrals among each other, which could affect the oral health of pregnant women. Thus, future computerized-based systems for ease of referral and educational programs should be implemented to deliver appropriate dental care for these specific patients.
